# Galvanic Apparatus for Clinical Use

**Published:** 1835

**Authors:** 


### VIII. Galvanic Apparatus for Clinical use.

One of the most cheap and convenient galvanic machines, for medicinal purposes, is that invented by Volta, and denominated *Couronne de Tasses*, on which some slight improvements have been made by Mr. Hentz and Dr. Locke. It consists of 20, 30, or more, very small tumblers, connected together by hanging a plate of copper and another of zinc, fastened by means of a narrow slip of the former metal, an inch long, and bent in the middle, so as to lie like a saddle over the edges of two of the glasses, being attached to moveable frame so as to be lifted on and off together. In this way the whole are formed into a battery, and the moment the glasses are filled with diluted acid, it becomes active. An apparatus of this kind, packed in a small box, may be had of T. Lawson and Sons, Main street, in this city.

